# Pregnancy in the Sickle Cell Disease and Fetomaternal Outcomes in Different Sickle cell Genotypes: A Systematic Review and Meta-Analysis

**DOI:** 10.4314/ejhs.v32i4.23

**Published:** 2022-07

**Authors:** Teamur Aghamolaei, Asiyeh Pormehr-Yabandeh, Zahra Hosseini, Nasibeh Roozbeh, Mahdieh Arian, Amin Ghanbarnezhad

**Affiliations:** 1 Cardiovascular Research Center, Hormozgan University of Medical Sciences, Bandar Abbas, Iran; 2 PhD Student in Health Education and Promotion, Social Determinants in Health Promotion Research Center, Hormozgan Health Institute, Hormozgan University of Medical Sciences, Bandar Abbas, Iran; 3 Social Determinants in Health Promotion Research Center, Hormozgan Health Institue, Hormozgan University of Medical Sciences, Bandar Abbas, Iran; 4 Mother and Child Welfare Research Center, Hormozgan University of Medical Sciences, Bandar Abbas, Iran; 5 Nursing and Midwifery Care Research Center, Mashhad University of Medical Sciences, Mashhad, Iran; 6 Social Determinants in Health Promotion Research Center, Hormozgan Health Institute, Hormozgan University of Medical Sciences, Bandar Abbas, Iran

**Keywords:** Anemia, Sickle Cell, Pregnancy, Fetus, Pregnancy Complications, Meta-Analysis

## Abstract

**Background:**

Pregnancy is a major concern among women with the sickle cell disease (SCD), and it is associated with increased adverse outcomes. The aim of the present meta-analysis is to report the fetomaternal outcomes in different sickle cell genotypes.

**Methods:**

In this systematic review and meta-analysis, a comprehensive search of databases and search engines such as PubMed, Scopus, Web of Science, ProQuest, Cochrane Library, Science Direct and Google Scholar were performed. Any observational studies that had compared at least one outcome such as maternal outcomes, fetal outcomes, and morbidity between two groups of pregnant women with different types of sickle cell genotypes and pregnant women without SCD were evaluated.

**Results:**

A total number of 9,827 pregnant women with SCD were examined. The results showed that pregnancy in SCD increased the risk of adverse outcomes for the mothers (including postpartum hemorrhage, prematurity, pregnancy-induced hypertension, pre-eclampsia, eclampsia, cesarean section, lower segment cesareansection, maternal death), fetus (including live births, low birth weight, intrauterine growth restriction, APGAR score at 5 min <7, stillbirth, neonatal death, perinatal mortality, acute fetal distress, intrauterine fetal death) and morbidity among the SCD(severe anemia, urinary tract infection, blood transfusion, painful crisis, acute chest syndrome, vaso-occlusive crises).

**Conclusion:**

According to the results of this meta-analysis, pregnancy in the SCD is associated with an increased risk of maternal outcomes, fetal outcomes, and morbidity among SCD patients with different genotypes. Pregnancy in sickle cell hemoglobinopathies needs careful multidisciplinary management and cautious caring so as to decrease maternal and fetal morbidity and mortality.

## Introduction

Sickle cell disease (SCD), caused by a mutation in the β-globin gene HBB, is the most inherited condition and is common in South African Sahara desert, South America, Central America, Saudi Arabia, India and Mediterranean countries (1). The predominant genotypes that give rise to SCD include Hb SS, Hb SC, Hb Sβ+-thalassemia and Hb Sβ0-thalassemia. Other rare forms include hemoglobin SD and hemoglobin SE (2). SCD is perceived as a global threat by World Health Organization (WHO) and about 5% of the world population and more than 7% of pregnant women worldwide suffer from hemoglobinopathies such as SCD (2–3). The adverse effects of this disease are serious infections, damage to vital body organs, brain stroke, renal disease, respiratory problems, bone marrow suppression, failure to thrive (FTT), cognitive disorder, delayed maturation in children and the high rate of maternal and fetal mortalities (3–4).

Many studies have shown that SCD is negatively associated with maternal health and prenatal conditions. The fetomaternal consequences of SCD are complicated. The main maternal complications of pregnancies complicated by SCD anemia, infection, vasoocclusive crisis, preeclampsia, preterm labor and the higher risk of the cesarean. The fetal problems that can affect perinatal outcomes are intrauterine growth restriction, premature birth, abnormal fetal heart rate and intrauterine fetal death. A high rate of maternal and fetal death has been reported in pregnant women with SCD than the healthy population (4–6). As already explained, this disease is accompanied by lifelong adverse effects and preterm mortality. Thus, a higher quality of taking care of people with SCD can improve survival and, thus, the number of fertile women (5). It has been previously shown that SCD can increase complications during pregnancy and in turn negatively influence pregnancy outcomes. However, the inherent heterogeneity of SCD pathophysiology in adjusting these studies can reduce trust in estimating the pregnancy risks of this disease (6–7). Insufficient information about the outcomes of this disease, with different genotypes, among pregnant women poses challenges in prenatal consultations and developing guideline recommendations based on the available evidence to provide comprehensive prenatal care services. Thus, the present systematic review and meta-analysis helps to explore the maternal and fetal outcomes of different genotypes of SCD taking into account the factors that might cause heterogeneity in the existing body of research evidence.

## Methods

The present study was conducted based on the preferred reporting items for systematic review and meta-analysis (PRISMA) checklist (8), but was not registered in the international prospective register of systematic reviews (PROSPERO) database and a public protocol does not exist. No ethical approval was sought for this systematic review. As this study is a systematic review of previously published studies, the need for ethics approval and patient informed consent was therefore waived. The components of structured question (PICO) were population (P): pregnant women with different types of sickle cell genotypes; and intervention (I): not required; comparison (C): with healthy pregnant women with HbAA; outcome (O): maternal outcomes, fetal outcomes, and morbidity in the SCD.

**Search strategy**: A comprehensive and regular search was done from inception to 14 December 2021, with the keywords (“Anemia, Sickle Cell” [MeSH]) AND (“Pregnancy” [MeSH] OR “Pregnant Women” [MeSH] OR “Fetus” [MeSH] OR “Obstetrics” [MeSH] OR “Pregnancy Complications” [MeSH]) without time and language restrictions in the following databases: Web of Science, Scopus, ProQuest, Cochrane Library, Science Direct, Medline, MEDLINE/PubMed and Google Scholar search engine. Also, the reference list of included studies was hand-searched for any relevant studies missing in the database searches. Prior to the search, it was decided that gray literature would not be searched as these studies are not peerreviewed and lacks quality control. Four weeks before we submitted the final manuscript to the journal, we performed an updated search on all specified databases.

**Eligibility criteria**: Any historical cohort, prospective cohort, retrospective cross-sectional, and retrospective case-control, observational case-control, descriptive studies with two comparators and descriptive cross-sectional studies, that had compared at least one outcome such as (maternal outcomes, fetal outcomes, and morbidity in the SCD) between two groups of pregnant women with different types of sickle cell genotypes and pregnant women without SCD were included in this systematic review and meta-analysis. Clinical trials, quasi-experimental studies, reviews, letter to editors, or case reports or those reporting outcomes in only one group or in non-pregnant women were excluded from this systematic review and meta-analysis.

**Figure 1:**
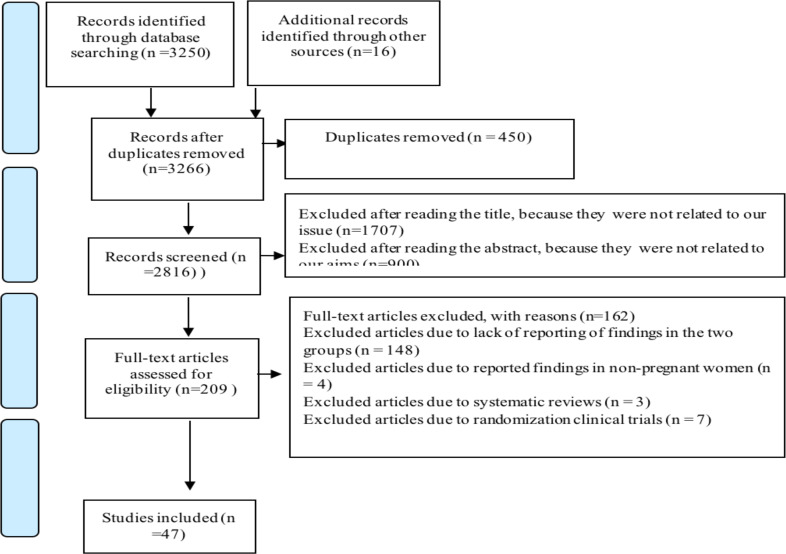
Flow diagram of the study

**Selection procedure**: EndNote X8 software was used to manage the included studies. Out of a total number of 3266 search, 450 texts were excluded due to duplication. Then, the titles of 2,816 texts were reviewed and 1,707 texts that were not related to the topic were excluded. The abstracts of 1,109 texts were reviewed and 900 texts that were not related to the aims were excluded. The full text of 209 studies was reviewed by two researchers (M.A & A.P) based on the inclusion and exclusion criteria. 148 studies were excluded due to the lack of a detailed reporting of findings in the two comparison groups; 4 were excluded due to the report of findings in non-pregnant women; 3 studies were excluded as they were systematic reviews, and 7 studies were so due to the use of randomization clinical trials. Finally, 47 studies were selected and they entered the quality evaluation stage (Figure 1).

**Quality assessment**: The Joanna Briggs Institute (JBI) Critical Appraisal 5-item Checklist was used for quality assessment of included studies (case-control studies and longitudinal cohort, or cross-sectional studies reporting the prevalence data) (9). The two authors independently reviewed each study based on the criteria in these checklists with the options of “Yes”, “No”, and “Unclear”. For each item, “Yes” had a score of 2, “Unclear” 1 and “No” had no score. The total scores of each study were considered as total scores. Quality classification of studies in this 5-item checklist was high (7–10), Moderate (3–6), and Weak (3>). Figure 2 shows a review of the biases of the reported studies. If there was any disagreements between two authors, it was resolved by consultation with the third author.

**Data extraction**: The data extraction was carried out independently by 2 authors (M.A. and A.P.) using a standard extraction form. The following information was extracted from each study: authors' names; year of publication; title; design; setting; the number of women in exposed and comparator groups; genotype; and outcomes. For each study, the required data were retrieved for the meta-analysis on the outcomes of interest: maternal outcomes such as premature rupture of membranes (PROM), postpartum hemorrhage (PPH), prematurity, pregnancy-induced hypertension (PIH), pre-eclampsia, eclampsia, gestational diabetes mellitus, cesarean section, lower segment caesarean section (LSCS), maternal death) and fetal outcomes. including (mean birth weight, live births, low birth weight (LBW), intrauterine growth restriction (IUGR).

APGAR score at 5 min <7, stillbirth, neonatal death, perinatal mortality, acute fetal distress (AFD), intra-uterine fetal death (IUFD), and morbidity among the SCD including (severe anemia, urinary tract infection (UTI), blood transfusion (BT), painful crisis, acute chest syndrome, vaso occlusive crises (VOC) were collected for each study. We obtained the gross national income per capita for each study from the World Bank data.

**Data synthesis and statistical analysis**: The analyses were pregnancy based. The main measure of the effect of maternal SCD on fetomaternal and pregnancy outcome was the unadjusted risk ratio, calculated from the given numbers of pregnancies and events. Separate comparisons were made for women with the total SCD, SCT, HbSS, HbSC. In each analysis, the reference group was women with SCT, HbAA, HbSC. If the number of studies in each comparison was at least 3, the analysis was done. Depending on the outcome under consideration, studies with no events in either arm were excluded. The pooled risk ratio was reported with 95% of confidence interval (95% CI). Besides, the randomized model was reported by 95% CI. A p-value < 0.05 was considered statistically significant. The Q statistic and the I^2^ index were used to assess the heterogeneity of the studies. The I^2^ index was used due to its accuracy to compensate for the lack of power (the Q statistic) in small sample sizes or increase the power in large sample sizes. In the I^2^ index, a value below 50% indicated a low variance in the studies. Moreover, a fixed effect model and the inverse variance method were used. Otherwise, instrumental variable (IV) heterogeneity method was used (10). Where substantial heterogeneity existed (by I^2^), mixed-effects analysis was used to test the study differences using the following variables: quality of study reporting; country gross national income (GNI), and the year of publication. The results are summarized as odds ratios. All statistical analyses were done in the Comprehensive Meta-analysis (version 2) and Rev-Man (version 5.3).

## Results

**Characteristics of the included studies**: In 47 studies published between 1977 and 2020, a total number of 9,827 pregnant women with SCD, 9,734,709 pregnant women with HbAA, 3,298 pregnant women with SCT, 730 pregnant women with HbSC, and 1,691 pregnant women with HbSS were examined. The pooled mean age of pregnant women in SCD according to 12 studies was 25.97 ± 4.8; HbAA according to 17 studies was 26.93 ± 5.49; SCT according to 4 studies was 26.59 ± 5.52; HbSC according to 5 studies was 27.12 ± 12.36 and HbSS according to 9 studies was 26.27± 9.24. The mean age of pregnant women did not show any statistically significant difference in any of the comparison groups (SCD vs HbAA) (SMD: -0.1, 95% CI: -0.2, 0.033), (SCD vs. SCT) (SMD: -0.23, 95% CI: - 0.5, 0.09), (HbSS vs. HbAA) (SMD: -0.08, 95% CI: -0.2, 0.04), (HbSS vs. HbSC) (SMD: -0.07, 95% CI: -0.2, 0.1). In this review, the total sample size of selected studies was variable from 50 to 8821321. The other information about the selected studies is listed in Table 1.

**Table 1:** Basic characteristics of the studies included in the meta-analysis

First author and year of publication	Country	country gross national income	Study design	Sample size	Quality of study reporting	
Nkwabong, 2020 [11]	Cameroon	Lower- middle-income	Historical cohort	120	Moderate	6
Galiba, 2020 [12]	Congo	Lower- middle-income	case-control	195	Moderate	7
Wellenstein, 2019 [13]	United States	High-income	Historical cohort	31840	High	9
Oppong, 2019 [14]	Ghana	Lower- middle-income	prospective cohort	266	High	8
Nwafor, 2019 [15]	Nigeria	Lower- middle-income	retrospective case-control	488	Moderate	7
Kumar Dora, 2019 [16]	India	Lower- middle-income	prospective observational	178	Moderate	6
Kose, 2019 [17]	India	Lower- middle-income	descriptive cross sectional	114	Moderate	5
Haseeb, 2019 [18]	Saudi Arabia	High-income	Historical cohort	902	High	8
Girish Mahajan, 2019 [19]	India	Lower- middle-income	Prospective, case control	180	High	9
Babah, 2019 [20]	Nigeria	Lower- middle-income	prospective case-control	100	High	10
Jyoti Kar, 2018 [21]	India	Lower- middle-income	prospective case control	2040	Moderate	7
Gaddikeri, 2017 [22]	India	Lower- middle-income	prospective	108	Moderate	7
Desai, 2017 [1]	India	Lower- middle-income	Case control	10036	High	8
DCouth, 2017 [23]	India	Lower- middle-income	retrospective observational	143	Moderate	7
Fouedjio, 2016 [24]	Cameroon	Lower- middle-income	retrospective	128	High	8
Elenga, 2016 [25]	Brazil	Upper-middle-income	retrospective	119	High	9
al-Jufairi, 2016 [26]	Bahrain	High-income	Retrospective Case- Control	370	High	8
Oteng-Ntim, 2015 [27]	United Kingdom	High-income	Prospective cohort	266496	High	9
Costa, 2015[28]	Brazil	Upper-middle-income	Prospective cohort	312	Moderate	6
Adama-Hondégla, 2015[29]	Togo	Low- middle-income	Descriptive and retrospective	226	High	10
Silva-Pinto, 2014 [30]	Brazil	Upper-middle-income	Historical cohort	61	High	8
Natu, 2014 [31]	India	Lower- middle-income	Historical cohort	579	High	9
Alayed, 2014 [32]	Canada	High-income	Historical cohort	8821321	High	8
Zia, 2013[33]	Saudi Arabia	High-income	Historical cohort	112	Moderate	6
Thame, 2013[34]	Jamaica	Upper-middle-income	Prospective cohort	82	Moderate	7
Muganyizi, 2013 [35]	Tanzania	Lower- middle-income	Historical cohort	155356	Moderate	6
Daigavane, 2013 [36]	India	Lower- middle-income	Prospective cohort	160	Moderate	6
Boulet, 2013 [37]	United States	High-income	Historical cohort	335348	High	8
Acharya, 2013 [38]	India	Lower- middle-income	Case control	50	High	8
Wilson, 2012[39]	Ghana	Lower- middle-income	Historical	1103	High	9
Al Kahtani, 2012 [40]	Saudi Arabia	High-income	Historical cohort	1176	Moderate	6
Nomura, 2010 [41]	Brazil	Upper-middle-income	Historical cohort	107	Moderate	6
Ngô, 2010[42]	France	High-income	Historical cohort	384	High	10
Barfield, 2010 [43]	United States	High-income	Historical cohort	115823	Moderate	7
Al Jama, 2009 [44]	Saudi Arabia	High-income	Historical cohort	755	Moderate	6
Afolabi, 2009 [45]	Nigeria	Lower- middle-income	Historical cohort	225	Moderate	7
Ashish, 2008 [46]	India	Lower- middle-income	Case control	224	Moderate	6
Thame, 2007 [47]	Jamaica	Upper-middle-income	Historical cohort	252	Moderate	5
Rajab, 2006 [48]	Bahrain	High-income	Historical cohort	662	Moderate	5
Serjeant, 2005 [49]	Jamaica	Upper-middle-income	Prospective cohort	535	Moderate	7
Serjeant, 2004 [50]	Jamaica	Upper-middle-income	Prospective cohort	120	Moderate	6
Sun, 2001[51]	United States	High-income	Historical cohort	509	Moderate	6
Al Mulhim, 2000[52]	Saudi Arabia	High-income	Historical cohort	198	Moderate	6
Balgir, 1997 [53]	India	Lower- middle-income	Historical cohort	190	Moderate	7
Howard, 1995 [54]	United Kingdom	High-income	Historical cohort	250	Moderate	7
Dare, 1992 [55]	Nigeria	Lower- middle-income	Historical cohort	142	Moderate	6
Blattner, 1977 [56]	United States	High-income	Prospective cohort	170	Moderate	6

**Association between SCD and maternal outcome**: The increased risk of PROM was not statistically significant in pregnant women with SCD vs HbAA based on 5 studies (RR: 1.1, 95% CI: 0.4, 2.9). The increased risk of PPH was statistically significant in pregnant women with SCD vs HbAA based on 7 studies (RR: 3.2, 95% CI: 1.2, 8). The increased risk of prematurity was statistically significant in pregnant women with SCD vs HbAA based on 7 studies (RR: 3.2, 95% CI: 1.2, 8) and HbSS vs. HbAA based on 16 studies (RR: 3.4, 95% CI: 2.1, 5.3) and HbSS vs HbSC based on 10 studies (RR: 1.6, 95% CI: 1.1, 2.3). The increased risk of PIH was statistically significant in pregnant women with HbSS vs. HbAA based on 6 studies (RR: 4.8, 95% CI: 1.6, 14.5). The increased risk of preeclampsia was statistically significant in pregnant women with SCD vs. AA based on 9 studies with (RR: 2.1, 95% CI: 1.5, 2.9), and also in the group with SCD vs SCT based on 3 studies (RR: 2.9, 95% CI: 1.3 6.6) and HbSS vs. HbAA based on 10 studies (RR: 2.8, 95% CI: 2.1, 3.8). The increased risk of eclampsia was statistically significant in pregnant women with SCD vs. HbAA based on 11 studies (RR: 2.7, 95% CI: 1.1, 6.4) and HbSS vs HbAA based on 6 studies (RR: 3.8, 95% CI: 1.4, 10). The increased risk of gestational diabetes mellitus (GDM) was not statistically significant in pregnant women with SCD vs. HbAA based on 3 studies (RR: 1.1, 95% CI: 0.8, 1.6) and SCD vs. SCT based on 3 studies (RR: 0.56, 95% CI: 0.1, 2.1). The increased risk of cesarean section was statistically significant in pregnant women with SCD vs. HbAA based on 6 studies (RR: 1.8, 95% CI: 1.5, 2.2) and HbSS vs. HbAA based on 9 studies (RR: 1.7, 95% CI: 1.4, 2.1). The increased risk of LSCS was statistically significant in pregnant women with HbSS vs HbAA based on 4 studies (RR: 3.7, 95% CI: 1.04, 13). The increased risk of maternal death was statistically significant in pregnant women with SCD vs. HbAA based on 9 studies with (RR: 7.2, 95% CI: 3.7, 19) and HbSS vs. HbAA based on 4 studies (RR: 37, 95% CI: 6, 222). The details of the analysis are reported in Table 2.

**Table 2:** Details of comparing maternal outcomes in women with SCD (based on different sickle cell genotypes) vs. women with no SCD

Maternal outcomes	Case-Control		Number of studies	Outcome among group1	Outcome among group2	95% confidence interval (95% CI)	P-Value	Heterogeneity			Egger
		
	Group1	Group2						df(Q)	P-Value	I Squared%	P-Value
**PROM**	SCD	AA	5	167/4837	336851 / 8818198	[0.4 – 2.9]	0.84	4	<0.0001	86	0.63
**PPH**	SCD	AA	7	119/2022	10422/337118	[1.2 – 8]	0.01	6	<0.0001	90	0.31
	SCD	SCT	6	33/292	29/310	[0.53 – 2.7]	0.64	5	0.051	53	0.84
	SCT	AA	3	12/162	42/2496	[0.36–24.1]	0.3	2	<0.0001	88	0.53
**Prematurity**	SCD	AA	17	910/4074	153911/873535	[1.6–2.3]	<0.0001	16	<0.0001	69	0.08
	SCT	AA	8	205/1335	2596/34407	[0.9–4.8]	0.08	7	<0.0001	94	0.53
	SS	AA	16	308/1116	50217/270758	[2.1–5.3]	<0.0001	15	<0.0001	90	0.73
	SS	SC	10	209/637	129/615	[1.1–2.3]	0.003	9	<0.0001	57	0.43
**PIH**	SCD	AA	4	57/722	711/9118	[0.7–3.6]	0.18	3	0.01	70	0.85
	SCD	SCT	3	13/245	143/1749	[0.3–1.8]	0.5	2	0.27	22	0.71
	SCT	AA	3	275/2579	4476/41174	[0.96–2.7]	0.06	2	<0.0001	91	0.3
	SS	AA	6	108/735	77/3592	[1.6–14.5]	0.004	5	<0.0001	92	0.59
	SS	SC	4	43/374	22/317	[0.5–3.7]	0.4	3	0.04	63	0.91
**Pre-eclampsia**	SCD	AA	9	575/5805	291915/8934162	[1.5–2.9]	<0.0001	8	<0.0001	75	0.15
	SCD	SCT	3	41 / 124	26 / 205	[1.3–6.6]	0.008	2	0.07	62	0.81
	SCT	AA	3	28/160	163/610	[0.4–1.9]	0.8	2	0.14	48	0.77
	SS	AA	10	114/987	78/2167	[2.1–3.8]	<0.0001	9	0.69	0	0.79
	SS	SC	9	77 / 699	56 / 664	[0.8–1.8]	0.3	8	0.32	13	0.15
**Eclampsia**	SCD	AA	11	267 / 7652	33941 / 12276629	[1.1–6.4]	0.025	10	<0.0001	94	0.64
	SCD	SCT	3	9 / 196	20 / 1731	[0.8–5.6]	0.09	2	0.64	0	0.73
	SS	AA	6	20 / 508	13 / 1354	[1.4–10]	0.006	5	0.22	25	0.03
	SS	SC	4	22 / 389	23 / 433	[0.7–2.4]	0.3	3	0.7	0	0.45
**GDM**	SCD	AA	3	52 / 799	91 / 1661	[0.8–1.6]	0.4	2	0.6	0	0.65
	SCD	SCT	3	5 / 148	10 / 137	[0.1–2.1]	0.4	2	0.3	18	0.54
**Cesarean section**	SCD	AA	16	1539 / 4279	178281 / 615649	[1.5–2.2]	<0.0001	15	<0.0001	91	0.06
	SCD	SCT	4	88 / 296	158 / 1805	[0.7–2.6]	0.2	3	<0.0001	86	0.73
	SS	AA	9	327 / 959	463 / 2163	[1.4–2.1]	<0.0001	8	0.01	57	0.35
	SS	SC	8	247 / 648	259 / 620	[0.8–1.8]	0.7	7	0.07	46	0.1
**LSCS**	SCD	AA	3	70 / 199	53 / 284	[0.9–4.5]	0.1	2	0.01	75	0.48
	SCD	SCT	3	68 / 177	61 / 221	[1–4.2]	0.06	2	0.02	72	0.14
	SCT	AA	3	40 / 140	49 / 2051	[0.93–41]	0.059	2	<0.0001	91	0.58
	SS	AA	4	82 / 201	109 / 2193	[1.04–13]	0.04	3	<0.0001	96	0.65
**Maternal death**	SCD	AA	9	40 / 5536	1579 / 8974283	[3.7–19	<0.0001	8	<0.0001	71	0.27
	SS	AA	4	12 / 179	0 / 2266	[6–222]	<0.0001	3	0.2	30	0.71

**PROM**: Premature rupture of membranes, **PPH**: Postpartum hemorrhage, **PIH**: Pregnancy-induced hypertension, **GDM**: Gestational diabetes mellitus, **LSCS**: Lower segment caesarean section, **SCD**: Sickle cell disease, **SCT**: Sickle cell trait

***Association between SCD and fetal outcome***: The decrease of live births was statistically significant in pregnant women with SCD vs. HbAA based on 5 studies (RR: 0.8, 95% CI: 0.7, 0.9) and HbSS vs. HbAA based on 5 studies (RR: 0.7, 95% CI: 0.7, 0.9). The increased risk of LBW was statistically significant in pregnant women with SCD vs. HbAA based on 11 studies (RR: 2.8, 95% CI: 1.6, 4.6), SCD vs. SCT based on 5 studies (RR: 1.7, 95% CI: 1.1, 2.5), SCT vs. HbAA based on 5 studies (RR: 3, 95% CI: 0.9, 9.3) and HbSS vs. HbAA based on 4 studies (RR: 11.8, 95% CI: 2.5, 54.5). The increased risk of IUGR was statistically significant in pregnant women with SCD vs AA based on 10 studies (RR: 2.3, 95% CI: 1.6, 3) and HbSS vs. HbAA based on 6 studies (RR: 7.3, 95% CI: 3.5, 15). The increased risk of Apgar score at 5 min <7 was statistically significant in pregnant women with SCD vs. HbAA based on 6 studies (RR: 1.9, 95% CI: 1.3, 2.6). The increased risk of stillbirth was statistically significant in pregnant women with SCD vs. HbAA based on 9 studies (RR: 5.7, 95% CI: 3, 10) and HbSS vs. HbAA based on 10 studies (RR: 10.8, 95% CI: 6.1, 10). The increased risk of neonatal death was statistically significant in pregnant women with SCD vs. HbAA based on 6 studies (RR: 2.2, 95% CI: 1.4, 4.5) and HbSS vs. HbAA based on 6 studies (RR: 2.9, 95% CI: 1.4, 5.8). The increased risk of perinatal mortality was statistically significant in pregnant women with SCD vs. HbAA based on 7 studies (RR: 3.3, 95% CI: 2.2, 5). The increased risk of AFD was statistically significant in pregnant women with SCD vs. HbAA based on 5 studies (RR: 3.2, 95% CI: 1.4, 7.1). The increased risk of IUFD was statistically significant in pregnant women with HbSS vs. HbAA based on 4 studies (RR: 5.4, 95% CI: 1.3, 22.7). The details of the analysis are reported in Table 3.

**Table 3:** Details of comparing fetal outcomes in women with SCD (based on different sickle cell genotypes) vs. women with no SCD

Fetal outcomes	Case-Control		Number of studies	Outcome among group1	Outcome among group2	Pooled risk ratio	95% confidence interval (95% CI)	P- Value	Heterogeneity			Egger
	
	Group 1	Group 2							df(Q)	P- Value	I- Square d	P- Value
**Live** **births**	SCD	AA	5	683 / 830	1618 / 1701	0.8	[0.7–0.9]	<0.0001	4	0.009	89	0.008
	SCD	SCT	3	88 / 107	113 / 130	0.94	[0.8–1.1]	0.46	2	0.06	64	0.56
	SS	AA	5	243 / 331	643 / 678	0.7	[0.7–0.9]	<0.0001	4	0.01	68	0.23
**Low** **birth** **weight**	SCD	AA	11	425/1944	35322 / 546396	2.8	[1.6–4.6]	<0.0001	10	<0.0001	96	0.19
	SCD	SCT	5	197 / 355	790 / 1924	1.7	[1.1–2.5]	0.005	4	0.001	75	0.83
	SCT	AA	5	806 / 1861	3873 / 10393	3	[0.9–9.3]	0.04	4	<0.0001	97	0.13
	SS	AA	4	58 / 193	1936 / 268448	11.8	[2.5–54.5]	0.001	3	<0.0001	97	0.23
	SS	SC	4	102 / 315	89 / 360	1	[0.5–3.2]	0.54	3	<0.0001	90	0.73
**IUGR**	SCD	AA	10	407 / 6359	157425 / 8942991	2.3	[1.6–3]	<0.0001	9	<0.0001	75	0.32
	SCD	SCT	4	46 / 377	65 / 1851	1.1	[0.4–3.3]	0.76	3	0.001	81	0.68
	SCT	AA	4	63 / 1872	151 / 10373	3.5	[0.9–13]	0.06	3	<0.0001	89	0.22
	SS	AA	6	102 / 587	54 / 3483	7.3	[3.5–15]	<0.0001	5	0.003	72	0.24
	SS	SC	4	61 / 324	36 / 392	1.4	[0.65–3]	0.35	3	0.06	58	0.8
**Apgar** **score at** **5 min** **<7**	SCD	AA	6	108 / 917	23103 / 156248	1.9	[1.3–2.6]	<0.0001	5	0.18	33	0.5
	SS	AA	5	22 / 345	27 / 585	1.7	[0.7–3.9]	0.16	4	0.12	45	0.1
	SS	SC	3	17 / 287	13 / 194	0.7	[0.3–1.5]	0.45	2	0.5	0	0.22
**Stillbirth**	SCD	AA	9	116/1403	12550/423892	5.7	[3–10]	<0.0001	8	0.02	55	0.53
	SCT	AA	4	9 / 280	4 / 302	2.2	[0.6–6.8]	0.18	3	0.96	0	0.95
	SS	AA	10	74 / 893	343 / 267740	10.8	[6.1–19]	<0.0001	9	0.83	0	0.2
	SS	SC	6	36 / 373	31 / 429	1.3	[0.7–2.5]	0.4	5	0.27	20	0.91
**Neonatal** **death**	SCD	AA	6	30 / 1120	19 / 1893	2.2	[1.4–4.5]	0.003	5	0.76	0	0.54
	SS	AA	6	23 / 732	13 / 1231	2.9	[1.4–5.8]	0.005	5	0.52	0	0.3
	SS	SC	4	18 / 295	16 / 355	1.8	[0.9–3.5]	0.06	3	0.84	0	0.13
**Perinatal** **mortality**	SCD	AA	7	95 / 1297	132 / 10866	3.3	[2.2–5]	<0.0001	6	0.37	6	0.16
	SS	AA	3	32 / 246	113 / 11173	8.6	[0.7–101]	0.08	2	<0.0001	90	0.82
**AFD**	SCD	AA	5	63 / 390	32 / 527	3.2	[1.4–7.1]	0.004	4	0.03	61	0.36
	SS	SC	4	32 / 236	28 / 322	1.5	[0.7–3.3]	0.26	3	0.15	42	0.99
**IUFD**	SCD	AA	5	78 / 4414	408 / 8817875	11.4	[0.9–141]	0.057	4	<0.0001	97	0.04
	SCT	AA	3	6 / 162	14 / 637	2.6	[1–7]	0.05	2	0.8	0	0.7
	SS	AA	4	7 / 139	2 / 307	5.4	[1.3–22.7]	0.01	3	0.4	0	0.21
	SS	SC	3	8 / 193	15 / 280	1.1	[0.16–8.2]	0.87	2	0.8	60	0.77

**IUGR**: Intrauterine growth restriction, **AFD**; Acute fetal distress, **IUFD**: Intra-uterine fetal death

Association between SCD and morbidity: The increased risk of severe anemia was statistically significant in pregnant women with HbSS vs. HbAA based on 4 studies (RR: 29, 95% CI: 4 , 203). The increase risk of UTI was statistically significant in pregnant women with SCD vs. HbAA based on 7 studies (RR: 2.1, 95% CI: 1.8, 2.4) and HbSS vs. HbAA based on 10 studies (RR: 5.1, 95% CI: 2.1, 12.4). The increased risk of BT was statistically significant in pregnant women with SCD vs. HbAA based on 7 studies (RR: 13, 95% CI: 6.2, 26), SCD vs. SCT based on 4 studies (RR: 11.5, 95% CI: 9.2, 15), HbSS vs. HbAA based on 4 studies (RR: 58, 95% CI: 10, 31), and HbSS vs. HbSC based on 8 studies (RR: 2.4, 95% CI: 1.7, 3.4). The increased risk of painful crisis was statistically significant in pregnant women with HbSS vs. HbAA based on 3 studies (RR: 117.1, 95% CI: 23.4, 586). The increased risk of acute chest syndrome was statistically significant in pregnant women with HbSS vs. HbAA based on 4 studies with (RR: 33, 95% CI: 7.5, 137.5). The increased risk of VOC was statistically significant in pregnant women with HbSS vs. HbAA based on 3 studies (RR: 47.6, 95% CI: 9.2, 245.2). The details of the analysis are reported in Table 4.

**Table 4:** Details of comparing morbidity women with SCD (based on different sickle cell genotypes) vs. women with no SCD

Morbidity among the sickle cell disease	Case-Control		Number of studies	Outcome among group1	Outcome among group2	Pooled risk ratio	95% confidence interval (95% CI)	P- Value	Heterogeneity			Egger
	
	Group 1	Group 2							df (Q)	P- Value	I- Squared	P- Value
**Severe**	SCD	SCT	3	21 / 141	14 / 189	1.2	[0.1–9]	0.8	2	0.002	83	0.26
**Anemia**	SS	AA	4	22 / 169	5 / 2196	29	[4–203]	0.001	3	0.02	69	0.03
**UTI**	SCD	AA	7	230 / 2146	14265 / 335402	2.1	[1.8–2.4]	<0.0001	6	0.62	0	0.44
	SS	AA	10	108 / 803	70 / 3918	5.1	[2.1–12.4]	<0.0001	9	<0.0001	82	0.89
	SS	SC	9	93 / 638	67 / 607	1	[0.7–1.5]	0.6	8	0.24	21	0.03
**BT**	SCD	AA	7	460 / 2126	3836 / 342613	13	[6.2–26]	<0.0001	6	<0.0001	94	0.67
	SCD	SCT	4	161 / 308	82 / 1872	11.8	[9.2–15]	<0.0001	3	0.8	0	0.3
	SCT	AA	3	75 / 1764	246 / 10286	4.3	[0.6–29.9]	0.13	2	<0.0001	87	0.4
	SS	AA	4	147 / 287	13 / 2319	58	[10–319]	<0.0001	3	<0.0001	88	0.24
	SS	SC	8	225 / 432	94 / 423	2.4	[1.7–3.4]	<0.0001	7	0.06	47	0.33
**Painful** **crisis**	SS	AA	3	115 / 460	0 / 880	117.1	[23.4–586]	<0.0001	2	0.7	0	0.31
	SS	SC	5	170 / 438	78 / 328	1.5	[0.8–2.8]	0.1	4	<0.0001	83	0.44
**Acute** **Chest** **Syndrome**	SS	AA	4	60 / 510	0 / 930	33	[7.9–137.5]	<0.0001	3	0.4	0	0.03
	SS	SC	4	27 / 242	22 / 300	1.6	[0.9–2.8]	0.1	3	0.9	0	0.7

**UTI**: Urinary tract infection, **BT**: Blood transfusion

Mixed-effects analysis: The mixed-effects analysis demonstrated that the year of publication, quality of study reporting, and GNI comprised the heterogeneity factors in comparing HbSS and HbAA groups for the outcomes of prematurity, PIH, LSCS, UTI, BT, in comparing SCD vs. HbAA for the cesarean section, live births, LBW, IUFD, BT), comparing SCD vs. SCT groups for the cesarean section, in (SCT vs HbAA) for (LSCS), and comparing HbSS vs HbSC groups for LBW. Also, the year of publication, and quality of reporting were found as the heterogeneity factors in comparing SCD vs. HbAA for PPH. The quality of study reporting, and GNI were found as the heterogeneity factors in the comparison of HbSS vs. HbAA for LBV. The year of publication of the study and GNI were found as the heterogeneity factors in comparing HbSS vs. HbAA for perinatal mortality. Also the quality of reporting the results reporting) was a heterogeneity factor for the outcome (IUGR) in comparing SCT vs. HbAA and SCD-SCT, and also for the BT outcome in comparing SCT vs. HbAA. The detailed effect of these factors on the study results is reported in Table 5.

**Table 5:** Mixed-effects analysis by (year of publication, quality of reporting, and country gross national income-GNI)

Variable	Subgroup	Odds ratios							
	
		Quality of study reporting			Country gross national income (GNI)				
		High	Moderate		High	Upper-middle	Lower-middle	Low	P
**PROM**	SCD-AA	-	-	-	0.8[0.6–1]	-	1.1[0–26]	-	0.11
**PPH**	SCD-AA	1.5[1.2–1.9]	10.53[1–104]	<0.0001	1.5[1.2–2]	-	4.8[0.8–28]	-	<0.0001
	SCT-AA	0.9[0.2–3.1]	5.7[0.36–88]	0.69	-	-	-	-	-
**Prematurity**	SCT-AA	1.1[0.6–1.8]	6.9[0.7–67]	0.37	1[0.8–1.3]	-	4.5[0.7–26]	-	0.54
	SS-AA	3.7[1.8–7]	6[2.3–16]	<0.0001	4.8[3.5–6.6]	4.5[1.3–15.7]	5.5[1.7–18]	-	<0.0001
**PIH**	SCT-AA	1.1[0.8–1.5]	20[5.7–75]	0.05	1.3[1.09–1.5]	4.2[0.2–83]	-	-	0.003
	SS-AA	5.2[2.9–9.3]	5.8[0.3–110]	<0.0001	3.5[2–5.9]	1.2[0.4–3.6]	18[2–111]	-	<0.0001
**Eclampsia**	SCD-AA	4.1[1.6–10]	1.4[0.9–2.2]	0.004	3.3[1–10]	-	2.2[1–4.6]	-	0.004
**Cesarean section**	SCD-AA	3.5[1.7–6.8]	1.8[1.3–2.4]	<0.0001	1.6[1.3–2.1]	3.6[2.3–5.9]	2.8[1.3–5.9]	-	<0.0001
	SCD-SCT	3.5[2.1–5.9]	1.1[0.5–2.1[	<0.0001	0.7[0.3–1.6]	1.9[0.75–5.1]	3.4[0.3–1.6]	-	<0.0001
**LSCS**	SCT-AA	1.9[0.56.9]	25[10.3–63]	<0.0001	8.6[0.9–76]	-	8.2[0.5–125]	-	0.01
	SS-AA	27[1.2–572]	6[0.7–50]	0.009	5[2–11]	1.1[0.6–2]	39[18–81]	-	<0.0001
**Live births**	SCD-AA	0.08[0.03–0.2]	0.1[0.06–0.3]	<0.0001	0.1[0.06–0.3]	0.3[0.2–0.5]	0.08[0.04–0.1]	-	<0.0001
**LBW**	SCD-AA	4.7[1–22]	2.9[2.1–3.9]	<0.0001	5[0.9–33]	-	3[2.3–3.8]	-	<0.0001
	SCT-AA	11.6[0.05–2196]	4.6[0.63–34]	0.08	-	-	-	-	-
	SS-AA	69[38–125]	17[0.95–319]	<0.0001	96[38–125]	-	17[0.9–319]	-	<0.0001
	SS-SC	2.2[1.3–3.8]	0.9[0.04–18]	0.002	3.8[2.2–6.8]	-	0.1[0.08–0.45]	2[1.1–3.7]	0.005
**IUGR**	SCD-SCT	0.5[0.1–2.2]	4[1.7–9.4]	0.02	3[0.6–16]	-	1.1[0.2–6]	-	0.2
	SCT-AA	2[0.6–6.5]	19[6.2–62]	<0.0001	-	-	-	-	-
**Perinatal mortality**	SS-AA	-	-	-	5.3[0.4–60]	-	12[0.4–358]	-	0.04
**IUFD**	SCD-AA	16[0.55–496]	6.5[2.6–16]	<0.0001	281[209–376]	-	5.2[2.2–12]	-	<0.0001
**Severe Anemia**	SCD-SCT	0.1[0.01–0.9]	3.5[0.5–22]	0.77	-	-	-	-	-
**UTI**	SS-AA	3.4[2.2–5]	10[1.9–56]	<0.0001	3.9[2–7]	-	6.7[1.8–25]	-	<0.0001
**BT**	SCD-AA	26[10–67]	28[3–236]	<0.0001	45[10–203]	-	15[2.3–104]	-	<0.0001
	SCT-AA	1[1.1–1.9]	11[1.9–62]	0.002	3.2[0.28–36]	-	5[0.3–68]	-	0.12
	SS-AA	38[14–101]	235[86–639]	<0.0001	236[32–1722]	-	82[12.6–545]	-	<0.0001
**Painful crisis**	SS-SC	3.2[0.5–21]	1.6[0.4–6]	0.1	3.4[1.2–9.5]	0.4[0.2–0.9]	2.3[1–5.2]	-	0.3

**PROM**: Premature rupture of membranes, **PPH**: Postpartum hemorrhage, **PIH**: Pregnancy-induced hypertension, **LSCS**: Lower segment caesarean section, **LBW**: Low birth weight, **IUGR**: Intrauterine growth restriction, **IUFD**: Intra-uterine fetal death, **UTI**: Urinary tract infection, **BT**: Blood transfusion

**Publication bias assessment:**In the present study, publication bias was estimated via the Egger test and the results are shown in Table 3–5. The graphical funnel plots were symmetrical in most zones and did not reveal any bias.

## Discussion

This systematic review and meta-analysis showed that pregnancy in SCD increased the risk of adverse outcomes for the mother (including PPH, prematurity, PIH, pre-eclampsia, eclampsia, cesarean section, LSCS, maternal death), and for the fetus (live births, LBW, IUGR, APGAR score at 5 min <7, stillbirth, neonatal death, perinatal mortality, AFD, IUFD) and morbidity among patients with the SCD (severe anemia, UTI, BT, painful crisis, acute chest syndrome, VOC). The results of the present study are consistent with the results of a meta-analysis conducted by Boafor et al. (2016). They reported that SCD was associated with IUGR (pooled OR 2.79, 95% CI: 1.85– 4.21), perinatal mortality (pooled OR 3.76, 95% CI: 2.34–6.06), and LBW (pooled OR 2.00, 95% CI: 1.42–2.83). SCD was also associated with an increased risk of pre-eclampsia (pooled OR 2.05, 95% CI: 1.47–2.85), maternal mortality (pooled OR 10.91, 95% CI: 1.83–65.11, P = 0.009), and eclampsia (pooled OR 3.02, 95% CI: 1.20–7.58) (4). The results of the present study are consistent with a meta-analysis by Oteng-Ntim et al. (2015). As these researchers reported, 21 studies (including 26,349 women with SCD and 26151746 women without SCD) were selected. Pregnancies in women with HbSS vs. HbAA were at an increased risk of maternal mortality (relative risk [RR], 5.98; 95% confidence interval [CI], 1.94 18.44), pre-eclampsia (RR, 2.43; 95% CI: 1.75–3.39), stillbirth (RR, 3.94; 95% CI: 2.60–5.96), preterm labor (RR, 2.21; 95% CI: 1.47–3.31), and small for gestational age infants (RR, 3.72; 95% CI: 2.32–5.98). A meta-regression revealed that in HbSS vs. HbSC, low gross national income, and high study quality were related to the increased RRs (6). In the present study, the mixed-effects analysis showed that in studies in lower-middle income group, the HbSS vs. HbAA genotype was associated with increased RRs in prematurity, PIH, LSCS, perinatal mortality, and UTI, and that HbAS vs. HbAA was associated with increased RRs in PIH and that SCD vs. HbAA was associated with increased RRs in PPH. Also, SCD vs. SCT genotype was associated with increased RRs in the cesarean section. In the studies in high income group, the HbSS vs. HbAA genotype and SCD vs. HbAA were associated with increased RRs in LBW, BT and HbSS vs. HbSC was associated with increased RRs in LBW. Despite the current developments OSD caring and management, as well as obstetrics, and neonatal medicine, there is still a close association between pregnancy complications and morbidity comorbidity and the increased risk of adverse fetomaternal outcomes [6]. Contrary to the existing developments in health care, especially in taking care of pregnant women over the past 4 decades, the maternal and fetal morbidity and mortality rate is high. The therapeutic interventions to improve pregnancy-related outcomes are restricted in women with SCD, particularly in those with the HbSS genotype (57).

In the present study, the mixed-effects analysis showed that in studies published in 2015 or later, the HbSS vs. HbAA genotype was associated with the increased RRs in prematurity, PIH, LSCS, perinatal mortality and UTI. The SCD vs. HbAA genotype was associated with increased RRs in PPH, cesarean section and LBW). The SCD vs. SCT genotype was associated with increased RRs in the cesarean section. The HbAS vs. HbAA genotype was associated with increased RRs in LSCS. In the studies published before 2015, the SCD vs. HbAA genotype was associated with increased RRs in eclampsia, IUFD, and BT. The HbSS vs. HbSC genotype was associated with increased RRs in LBW. The HbSS vs. HbAA genotype was associated with increased RRs in BT and the HbAS vs. HbAA genotype. Totally, the adverse outcomes in pregnancy were worse and more prevalent in pregnant women with SCD vs. those without SCD. This study reports that pregnancy complications are more frequent in HbSS than other genotypes. These findings are matched with the reports of several studies (6, 15, 57, 58). The outcomes of pregnancy in the HbSS genotype were worse than HbAA and HbAS. Also, fetomaternal outcomes were worse in HbAS when compared with HbAA. The decreased risk of adverse pregnancy outcome in women with HbSC is matched with the manner of the HbSC genotype. This genotype is frequently benign and may not be recognized until later in adult life (15). The results of a study in Brazil indicated that in women with SCD, the HbSS genotype was associated with a higher frequency of blood transfusion. Also, Sβ-thalassemia was associated with a higher frequency of postpartum adverse events (59). In this study, HbSC women had better pregnancy outcomes. However, the incidence of sickle cell-related complications did not differ between women with the HbSS and HbSC genotype. Therefore, it is not yet possible to predict SC patients who may develop severe complications in pregnancy and it is an acceptable practice to assess all pregnancies in SCD expecting a baby in the hospital. However, Malinowski et al. suggested that early identification of women with SCD at high risk of maternal and fetal pregnancy adverse outcomes can be predicted using routine clinical and laboratory data (60). There are some limitations of this systematic review which should be noted. First, this systematic review was not registered on prospective registration systems for systematic reviews. Prospective registration could improve the quality of a systematic review and increase confidence in the findings. However our results were reported according PRISMA statement in order to minimize possible bias. Second, we did not searched the grey literatures and may could not identify any unpublished research. Like with any systematic review, there is always the risk of publication bias as studies with negative results are usually not published.

According to the results of this meta-analysis, pregnancy in the SCD is associated with an increased risk of maternal outcomes, fetal outcomes, and morbidity among patients with the SCD. This condition requires careful multidisciplinary management and cautious caring so as to decrease maternal and fetal morbidity and mortality. Therefore, accurate and timely follow-up and monitoring of these pregnancies with a multidisciplinary team comprised of a hematologist, an obstetrician, and a pediatrician is essential. Raising patients' awareness and educating them through communication sessions and a timely screening of complications for women with the SCD are essential to decrease the associated risks.

## References

[1] Desai G, Anand A, Shah P, Shah S, Dave K, Bhatt H (2017). Sickle cell disease and pregnancy outcomes: a study of the community-based hospital in a tribal block of Gujarat, India. J Health Popul Nutr.

[2] Saraf SL, Molokie RE, Nouraie M, Sable CA, Luchtman-Jones L, Ensing GJ (2014). Differences in the clinical and genotypic presentation of sickle cell disease around the world. Paediatr Respir Rev.

[3] Al-Azri MH, Al-Belushi R, Al-Mamari M, Davidson R, Mathew AC (2016). Knowledge and Health Beliefs Regarding Sickle Cell Disease Among Omanis in a Primary Healthcare Setting: Cross-sectional study. Sultan Qaboos Univ Med J.

[4] Olatona F, Odeyemi K, Onajole A, Asuzu MC (2012). Effects of Health Education on Knowledge and Attitude of Youth Corps Members to Sickle Cell Disease and its Screening in Lagos State. J Community Med Health Educ.

[5] Jain D, Atmapoojya P, Colah R, Lodha P (2019). Sickle Cell Disease and Pregnancy. Mediterr J Hematol Infect Dis.

[6] Oteng-Ntim E, Meeks D, Seed PT, Webster L, Howard J, Doyle P (2015). Adverse maternal and perinatal outcomes in pregnant women with sickle cell disease: systematic review and meta-analysis. Blood.

[7] Sundd P, Gladwin MT, Novelli EM (2019). Pathophysiology of Sickle Cell Disease. Annu Rev Pathol.

[8] Moher D, Shamseer L, Clarke M, Ghersi D, Liberati A, Petticrew M (2015). Preferred reporting items for systematic review and meta-analysis protocols (PRISMA-P) 2015 statement. Syst Rev.

[9] Munn Z, Tufanaru C, Aromataris E (2014). JBI's systematic reviews: data extraction and synthesis. Am J Nurs.

[10] Higgins JP, Thompson SG (2002). Quantifying heterogeneity in a meta-analysis. Stat Med.

[11] Nkwabong E, Ngoundjou Dongmo P, Tayou C, Nana Njamen T (2020). Outcome of pregnancies among women with sickle cell disease. J Matern Fetal Neonatal Med.

[12] Galiba Atipo Tsiba FO, Itoua C, Ehourossika C, Ngakegni NY, Buambo G, Potokoue Mpia NSB (2020). Pregnancy Outcomes among Patients with Sickle Cell Disease in Brazzaville. Anemia.

[13] Wellenstein WL, Sullivan S, Darbinian J, Ritterman Weintraub ML, Greenberg M (2019). Adverse Pregnancy Outcomes in Women with Sickle Cell Trait. AJP Rep.

[14] Oppong SA, Asare EV, Olayemi E, Boafor T, Dei-Adomakoh Y, Swarry-Deen A (2019). Multidisciplinary care results in similar maternal and perinatal mortality rates for women with and without SCD in a low-resource setting. Am J Hematol.

[15] Nwafor J, Ugoji D, Ibo C, Onwe B, Onuchukwu V, Obi C (2019). Pregnancy Outcome among Women with Sickle Cell Disease in a Tertiary Health Institution in Abakaliki: A Retrospective Case-Control Study. Int J Clin Med.

[16] Kumar Dora S, Dandapat A, Pande B, Bhoi G, Tiwari B (2019). A Prospective Study to Compare the Maternal and Fetal Outcomes among Sickle Cell Disease and Trait Women. J South Asian Feder Obst Gynae.

[17] Kose V, Kose S (2019). Pregnancy outcome in women with sickle cell disease/trait in a tertiary care hospital Nagpur, Maharashtra India: a descriptive cross sectional study. Int J Reprod Contracept Obstet Gynecol.

[18] Haseeb YA, Al Qahtani NH (2019). Outcome of Pregnancy in Saudi Women with Sickle Cell Disease Attending the Tertiary Care University Hospital in Eastern Province of Saudi Arabia. Afr J Reprod Health.

[19] Girish Mahajan A, Nagaria T, Kishore R (2019). Pregnancy in Sickle Cell Disease is a Very High-Risk Situation: A Case Control Study. J Med Sci Clin Res.

[20] Babah OA, Aderolu MB, Oluwole AA, Afolabi BB (2019). Towards zero mortality in sickle cell pregnancy: A prospective study comparing haemoglobin SS and AA women in Lagos, Nigeria. Niger Postgrad Med J.

[21] Jyoti Kar T, Baru L, Bhoi G, Patnaik R (2018). Fetomaternal outcome in sickle cell hemoglobinopathy. Glob J Res Anal.

[22] Gaddikeri A, Pajai S, Rathod A (2017). Pregnancy and Its Outcome in Sickle Cell Hemoglobinopathies: A Study of Central India. J South Asian Feder Obst Gynae.

[23] D'Couth S, Kalam S (2017). Fetomaternal outcome in sickle cell hemoglobinopathy in a tertiary care centre of North Kerala, India. Int J Reprod Contracept Obstet Gynecol.

[24] Fouedjio JH, Fouelifack FY, Fouogue JT, Ngoufack G, Fouelifa LD, Mbu R (2016). Maternal and Foetal Outcomes of Pregnancy with Homozygous Sickle Cell Disease: A Case - Control Study at the Yaounde Central Hospital, Cameroon. Women Heal Int.

[25] Elenga N, Adeline A, Balcaen J, Vaz T, Calvez M, Terraz A (2016). Pregnancy in Sickle Cell Disease Is a Very High-Risk Situation: An Observational Study. Obstet Gynecol Int.

[26] Al Jufairi ZA, Al Aradi FA, Sandhu AK (2016). Pregnancy outcome of sickle cell disease women. Bahrain Medical Bulletin. Bahrain Med Bull.

[27] Oteng-Ntim E, Ayensah B, Knight M, Howard J (2015). Pregnancy outcome in patients with sickle cell disease in the UK--a national cohort study comparing sickle cell anaemia (HbSS) with HbSC disease. Br J Haematol.

[28] Costa VM, Viana MB, Aguiar RA (2015). Pregnancy in patients with sickle cell disease: maternal and perinatal outcomes. J Matern Fetal Neonatal Med.

[29] Adama-Hondégla A, Aboubakari A, Logbo-Akey K, Fiagnon K, Bassowa A, Akpadza K (2015). Delivery Outcome in Women with Major Sickle Cell Syndrome: A Comparative Study of the Homozygous Forms “SS” versus the Heterozygous “SC”. Open J Obstet Gynecol.

[30] Silva-Pinto AC, de Oliveira Domingues Ladeira S, Brunetta DM, De Santis GC, de Lucena Angulo I, Covas DT (2014). Sickle cell disease and pregnancy: analysis of 34 patients followed at the Regional Blood Center of Ribeirão Preto, Brazil. Rev Bras Hematol Hemoter.

[31] Natu N, Khandelwal S, Kumar R, Dave A (2014). Maternal and perinatal outcome of women with sickle cell disease of a tribal population in Central India. Hemoglobin.

[32] Alayed N, Kezouh A, Oddy L, Abenhaim HA (2014). Sickle cell disease and pregnancy outcomes: population-based study on 8.8 million births. J Perinat Med.

[33] Zia S, Rafique M (2013). Comparison of pregnancy outcomes in women with sickle cell disease and trait. J Pak Med Assoc.

[34] Thame MM, Osmond C, Serjeant GR (2013). Fetal growth in women with homozygous sickle cell disease: an observational study. Eur J Obstet Gynecol Reprod Biol.

[35] Muganyizi PS, Kidanto H (2013). Sickle cell disease in pregnancy: trend and pregnancy outcomes at a tertiary hospital in Tanzania. PLoS One.

[36] Daigavane MM, Jena RK, Kar TJ (2013). Perinatal outcome in sickle cell anemia: a prospective study from India. Hemoglobin.

[37] Boulet SL, Okoroh EM, Azonobi I, Grant A, Craig Hooper W (2013). Sickle cell disease in pregnancy: maternal complications in a Medicaid-enrolled population. Matern Child Health J.

[38] Acharya A, AKriplani A, Hariharan C (2013). Study of perinatal outcome in pregnancy with sickle cell disease. Int J Biol Med Res.

[39] Wilson NO, Ceesay FK, Hibbert JM, Driss A, Obed SA, Adjei AA (2012). Pregnancy outcomes among patients with sickle cell disease at Korle-Bu Teaching Hospital, Accra, Ghana: retrospective cohort study. Am J Trop Med Hyg.

[40] Al Kahtani MA, AlQahtani M, Alshebaily MM, Abd Elzaher M, Moawad A, Aljohani N (2012). Morbidity and pregnancy outcomes associated with sickle cell anemia among Saudi women. Int J Gynaecol Obstet.

[41] Nomura RM, Igai AM, Tosta K, da Fonseca GH, Gualandro SF, Zugaib M (2010). Maternal and perinatal outcomes in pregnancies complicated by sickle cell diseases. Rev Bras Ginecol Obstet.

[42] Ngô C, Kayem G, Habibi A, Benachi A, Goffinet F, Galactéros F (2010). Pregnancy in sickle cell disease: maternal and fetal outcomes in a population receiving prophylactic partial exchange transfusions. Eur J Obstet Gynecol Reprod Biol.

[43] Barfield WD, Barradas DT, Manning SE, Kotelchuck M, Shapiro-Mendoza CK (2010). Sickle cell disease and pregnancy outcomes: women of African descent. Am J Prev Med.

[44] Al Jama FE, Gasem T, Burshaid S, Rahman J, Al Suleiman SA, Rahman MS (2009). Pregnancy outcome in patients with homozygous sickle cell disease in a university hospital, Eastern Saudi Arabia. Arch Gynecol Obstet.

[45] Afolabi BB, Iwuala NC, Iwuala IC, Ogedengbe OK (2009). Morbidity and mortality in sickle cell pregnancies in Lagos, Nigeria: a case control study. J Obstet Gynaecol.

[46] Ashish K, Raseswari P, Pruthviraj S (2008). Perinatal outcome in pregnancy with sickle cell anemia. J Obstet Gynecol India.

[47] Thame M, Lewis J, Trotman H, Hambleton I, Serjeant G (2007). The mechanisms of low birth weight in infants of mothers with homozygous sickle cell disease. Pediatrics.

[48] Rajab KE, Issa AA, Mohammed AM, Ajami AA (2006). Sickle cell disease and pregnancy in Bahrain. Int J Gynaecol Obstet.

[49] Serjeant GR, Hambleton I, Thame M (2005). Fecundity and pregnancy outcome in a cohort with sickle cell-haemoglobin C disease followed from birth. BJOG.

[50] Serjeant GR, Loy LL, Crowther M, Hambleton IR, Thame M (2004). Outcome of pregnancy in homozygous sickle cell disease. Obstet Gynecol.

[51] Sun PM, Wilburn W, Raynor BD, Jamieson D (2001). Sickle cell disease in pregnancy: twenty years of experience at Grady Memorial Hospital, Atlanta, Georgia. Am J Obstet Gynecol.

[52] Al Mulhim K (2000). Pregnancy in sickle cell disease in the Al Hassa Region of Saudi Arabia. Ann Saudi Med.

[53] Balgir RS, Dash BP, Das RK (1997). Fetal outcome and childhood mortality in offspring of mothers with sickle cell trait and disease. Indian J Pediatr.

[54] Howard RJ, Tuck SM, Pearson TC (1995). Pregnancy in sickle cell disease in the UK: results of a multicentre survey of the effect of prophylactic blood transfusion on maternal and fetal outcome. Br J Obstet Gynaecol.

[55] Dare FO, Makinde OO, Faasuba OB (1992). The obstetric performance of sickle cell disease patients and homozygous hemoglobin C disease patients in Ile-Ife, Nigeria. Int J Gynaecol Obstet.

[56] Blattner P, Dar H, Nitowsky HM (1977). Pregnancy Outcome in Women With Sickle Cell Trait. JAMA.

[57] Sousa VT, Ballas SK, Leite JM, Olivato MCA, Cancado RD (2021). Maternal and perinatal outcomes in pregnant women with sickle cell disease: an update. Hematol Transfus Cell Ther.

[58] Modi RS, Patel SS, Modi DA, Talesara H (2021). Fetomaternal outcome in sickle cell disease in a tertiary care centre. Int J Reprod Contracept Obstet Gynecol.

[59] Silva FAC, Ferreira ALCG, Hazin-Costa MF, Dias MLG, Araújo AS, Souza AI (2018). Adverse clinical and obstetric outcomes among pregnant women with different sickle cell disease genotypes. Int J Gynaecol Obstet.

[60] Malinowski AK, Kuo KHM, Tomlinson GA, Palcu P, Ward R, Shehata N (2021). Distinct maternal and fetal pregnancy outcomes in women with sickle cell disease can be predicted using routine clinical and laboratory data. Br J Haematol.

